# Examining the Effect of Kindlin-3 Binding Site Mutation on LFA-1-ICAM-1 Bonds by Force Measuring Optical Tweezers

**DOI:** 10.3389/fimmu.2021.792813

**Published:** 2022-01-26

**Authors:** Craig McDonald, Vicky L. Morrison, David McGloin, Susanna Carola Fagerholm

**Affiliations:** ^1^ SUPA, School of Science and Engineering, University of Dundee, Dundee, United Kingdom; ^2^ School of Medicine, University of Dundee, Dundee, United Kingdom; ^3^ School of Electrical and Data Engineering, University of Technology Sydney, Sydney, NSW, Australia

**Keywords:** LFA-1, kindlin-3, T cell, bond strength, ICAM-1

## Abstract

Integrins in effector T cells are crucial for cell adhesion and play a central role in cell-mediated immunity. Leukocyte adhesion deficiency (LAD) type III, a genetic condition that can cause death in early childhood, highlights the importance of integrin/kindlin interactions for immune system function. A TTT/AAA mutation in the cytoplasmic domain of the *β*2 integrin significantly reduces kindlin-3 binding to the *β*2 tail, abolishes leukocyte adhesion to intercellular adhesion molecule 1 (ICAM-1), and decreases T cell trafficking *in vivo*. However, how kindlin-3 affects integrin function in T cells remains incompletely understood. We present an examination of LFA-1/ICAM-1 bonds in both wild-type effector T cells and those with a kindlin-3 binding site mutation. Adhesion assays show that effector T cells carrying the kindlin-3 binding site mutation display significantly reduced adhesion to the integrin ligand ICAM-1. Using optical trapping, combined with back focal plane interferometry, we measured a bond rupture force of 17.85 ±0.63 pN at a force loading rate of 30.21 ± 4.35 pN/s, for single integrins expressed on wild-type cells. Interestingly, a significant drop in rupture force of bonds was found for TTT/AAA-mutant cells, with a measured rupture force of 10.08 ± 0.88pN at the same pulling rate. Therefore, kindlin-3 binding to the cytoplasmic tail of the *β*2-tail directly affects catch bond formation and bond strength of integrin–ligand bonds. As a consequence of this reduced binding, CD8+ T cell activation *in vitro* is also significantly reduced.

## 1 Introduction

The ability of leukocytes to traffic into tissues and to make contact with other cells depends chiefly on *β*2 integrins, which are expressed exclusively on leukocytes (1). The *αLβ*2 integrin, commonly called leukocyte function-associated antigen 1 (LFA-1), is the most abundant and widespread in expression of the four members of the *β*2 family (1, 2). LFA-1 is expressed by all leukocytes and binds to members of the intercellular adhesion molecule (ICAM) family (1). This integrin mediates the firm adhesion of leukocytes to the endothelial cells surrounding blood vessels and is necessary for leukocyte extravasation at the inflammatory site (3). The adhesion of murine effector T cells, generated *in vitro*, which play a central role in cell-mediated immunity, to ICAM-1 is completely dependent on *β*2 integrins (4).

The fundamental importance of *β*2 integrins is highlighted in the pathologies of several diseases and genetic syndromes, such as leukocyte adhesion deficiency (LAD) (1). LAD can be divided into three different subtypes, LAD-I–III, depending on the causative mutation (5), with types I and III being the most common. Patients who suffer from LAD-I have mutations that lead to a deficiency or absence of *β*2 integrin expression (1). Therefore, patients suffer from recurrent bacterial and fungal infections, delayed wound healing, and periodontitis (1). LAD-III, a variant of LAD-I, causes patients to present with similar symptoms to patients with LAD-I but with the added complications of a Glanzmann-type bleeding disorder and, in some cases, osteopetrosis (1). Mutations in the signalling protein kindlin-3 have been found to be the cause of LAD-III, thus preventing integrins from becoming fully activated, leading to deficiencies in T cell adhesion and homing (1, 6, 7).

Interaction of kindlin-3 with the *β*2 integrin in effector T cells is necessary for integrin-mediated adhesion to endothelial cells (6). Through mutating the TTT motif in the *β*2 integrin cytoplasmic domain, Morrison et al. generated a knock-in (KI) mouse where integrin binding to ICAM-1, T cell trafficking into lymph nodes and inflammatory sites, and T cell activation were severely diminished (7–9). The TTT/AAA mutation abolishes the interaction between integrin and kindlin-3 (7). However, the mechanism by which kindlin-3 affects integrin binding to ligand is still under debate, and it is currently unclear whether integrin/kindlin interactions affect integrin affinity, clustering, or other events involved in cellular adhesion (10–12).

In this paper, we directly assess the effect of mutation of the kindlin-3 binding site in LFA-1 on LFA-1/ICAM-1 bonds. We present an optical-tweezer-based approach for the quantification of the LFA-1–ICAM-1 bond rupture force. Primary murine effector T cells from wild-type and TTT/AAA-beta2-integrin KI mice were allowed to bind to ligand-coated silica beads, and the rupture force of LFA-1–ICAM-1 bonds at a specific pulling rate was measured. The results reveal that mutation of the kindlin-3 binding site in the *β*2-integrin results in a significantly lower rupture force of integrin–ligand bonds, indicating deficiency in integrin–ligand catch bond formation and resulting in lower bond strength. In addition, we show that this results in reduced cellular adhesion to coated ICAM-1 and in reduced CD8+ T cell activation *in vitro*. These findings thereby shed light on the role of kindlin-3 in regulation of LFA-1 function in T cells.

## 2 Materials and Methods

### 2.1 Effector T Cell Generation

Effector T cells were generated as in Lek et al. (4). Briefly, splenocytes from either wild-type (WT) or TTT/AAA- *β*2 integrin knock-in (KI) mice were activated with 0.5 *μ* g/ml anti-CD3 (clone 2C11, R&D Systems, Minneapolis, MN, USA) together with 20 ng/ml IL-2 (R&D Systems). After 2 days, cells were washed free of activating agent and then maintained in 20 ng/ml IL-2 to be used for experimental purposes on days 6–8 of the culture. Throughout the culture, cells were passaged every 2 days, as well as the day before use in an experiment, to ˜1 x 10^6^ cells per ml.

### 2.2 Static Adhesion Assay

Adhesion assays of effector T cells to coated ICAM-1 were performed as in Lek et al. (4).

### 2.3 T Cell Activation

CD8+ T cells were isolated using a MACS kit. Purified cells were suspended at 1 million cells/ml in medium and activated with 2.5 *μ*g/ml soluble anti-CD3 antibody (clone 2C11, R&D systems) for the indicated times, followed by flow cytometry to detect the expression of activation markers or ELISA to detect the expression of cytokines, as previously (7).

### 2.4 ICAM-1 Bead Coating

Silica beads with a diameter of 2.56 *μ*m were coated with 2 *μ*g/ml ICAM-1 (R&D Systems). A large excess of ICAM-1 relative to beads was used to ensure full coating of the beads. Beads were washed twice in 1 ml PBS, by resuspending the beads in 1 ml PBS followed by centrifugation at 13,000 rpm for 1 min in an Eppendorf centrifuge and collecting the beads. After this, beads were resuspended in 500 *μ*l of PBS with 2 *μ*g/ml ICAM-1 and incubated, on rotation, at 4°C for at least 1 h. After incubation, beads were washed 3 times in PBS as above, to remove any excess ICAM-1 from solution.

### 2.5 Sample Preparation

Samples were prepared by mixing 15 *μ*l of cells with 5 *μ*l of silica beads. The mixture was placed between two microscope coverslips, separated by a 100-μm-thick vinyl spacer. Cells were left to settle for 15–30 min at room temperature before adhesion experiments. This allowed cells to settle on the coverslip, ensuring they were neither pushed away by the bead, nor attracted to the optical trap.

### 2.6 Experimental System

**Figure 1:**
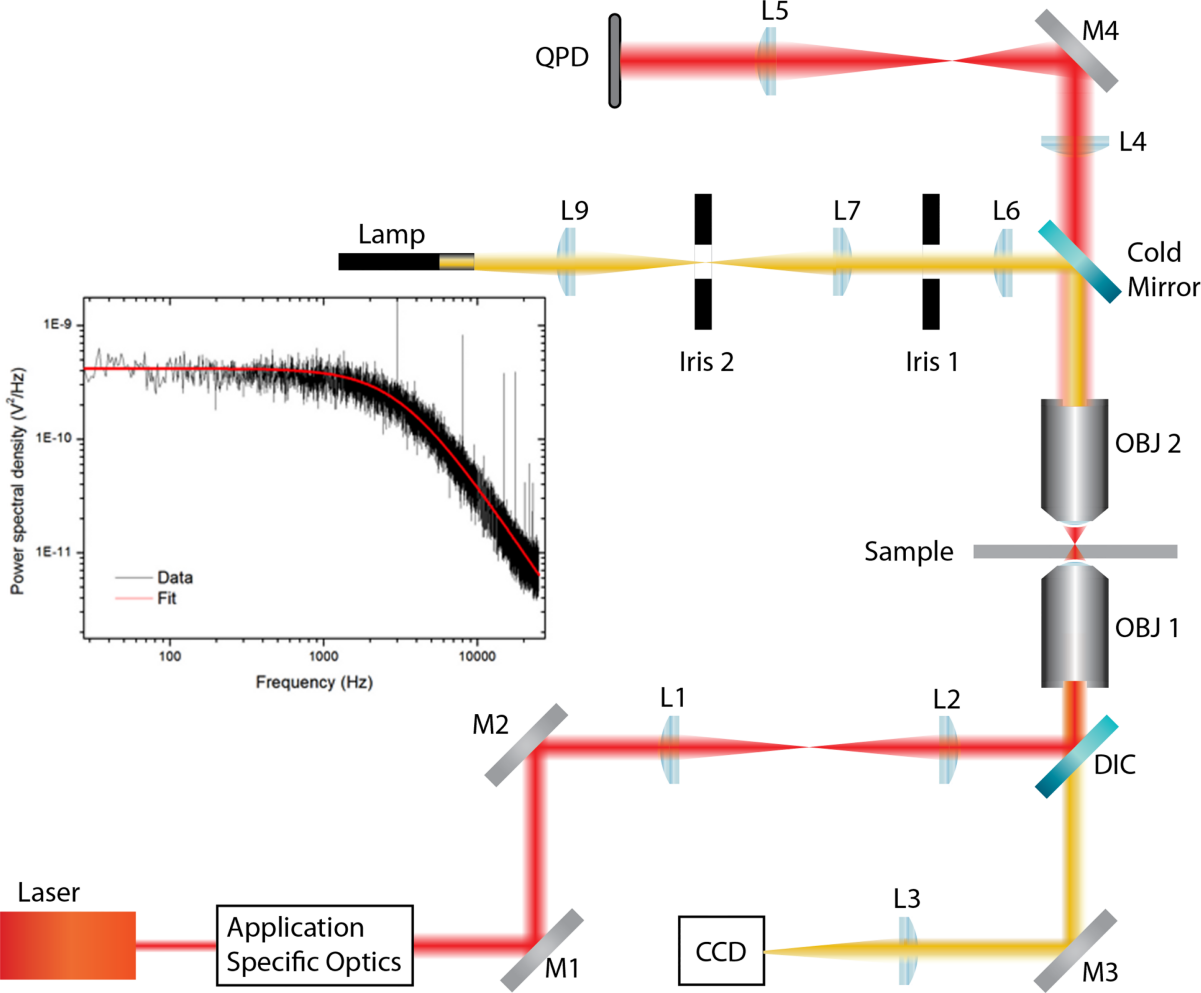
Force measuring optical tweezer system used for static adhesion assays. OBJ 1, Nikon 1.25 NA 100x oil immersion objective; OBJ 2, Mitutoyo 0.55NA 100x long working distance objective. DIC, dichroic mirror; QPD, quadrant photodiode; L, lens; M, mirror. Application-specific optics refer to expansion and beam steering optics. The sample was placed on a Thorlabs MAX302/M NanoMax piezoelectric stage, which was connected to a Thorlabs MDT630A 3-Axis piezo controller.

A force measuring optical tweezers (**Figure 1**) was constructed by expanding the beam from a 1.5-W (maximum output) 1,064-nm laser (Laser Quantum ventus 1064, Laser Quantum, Stockport, United Kingdom) to overfill the back aperture of a Nikon 1.25 NA 100x oil immersion objective (Spectra Services, Ontario, NY, USA). Samples were placed on a Thorlabs MAX302/M NanoMax piezoelectric stage (Thorlabs, Newton, NJ, USA), which was connected to a Thorlabs MDT630A 3-Axis piezo controller. The trap was imaged, *via* a Mitutoyo 0.55 NA 100x long working distance objective, onto a quadrant photodiode (QPD). The 2-mm-diameter InGaAs QPD (Hamamatsu, G6849, Hamamatsu Photonics, Hamamatsu, Japan) was used in back focal plane interferometry mode (13) and was connected to custom-built transimpedance amplifiers, which in turn were connected to a National Instruments SCB-68A connector block (National Instruments, Austin, TX, USA). Signals were collected *via* a National Instruments PCI-6250 data acquisition card and analysed using an in-house LabVIEW program.

### 2.7 Adhesion Measuring Program

**Figure 2:**
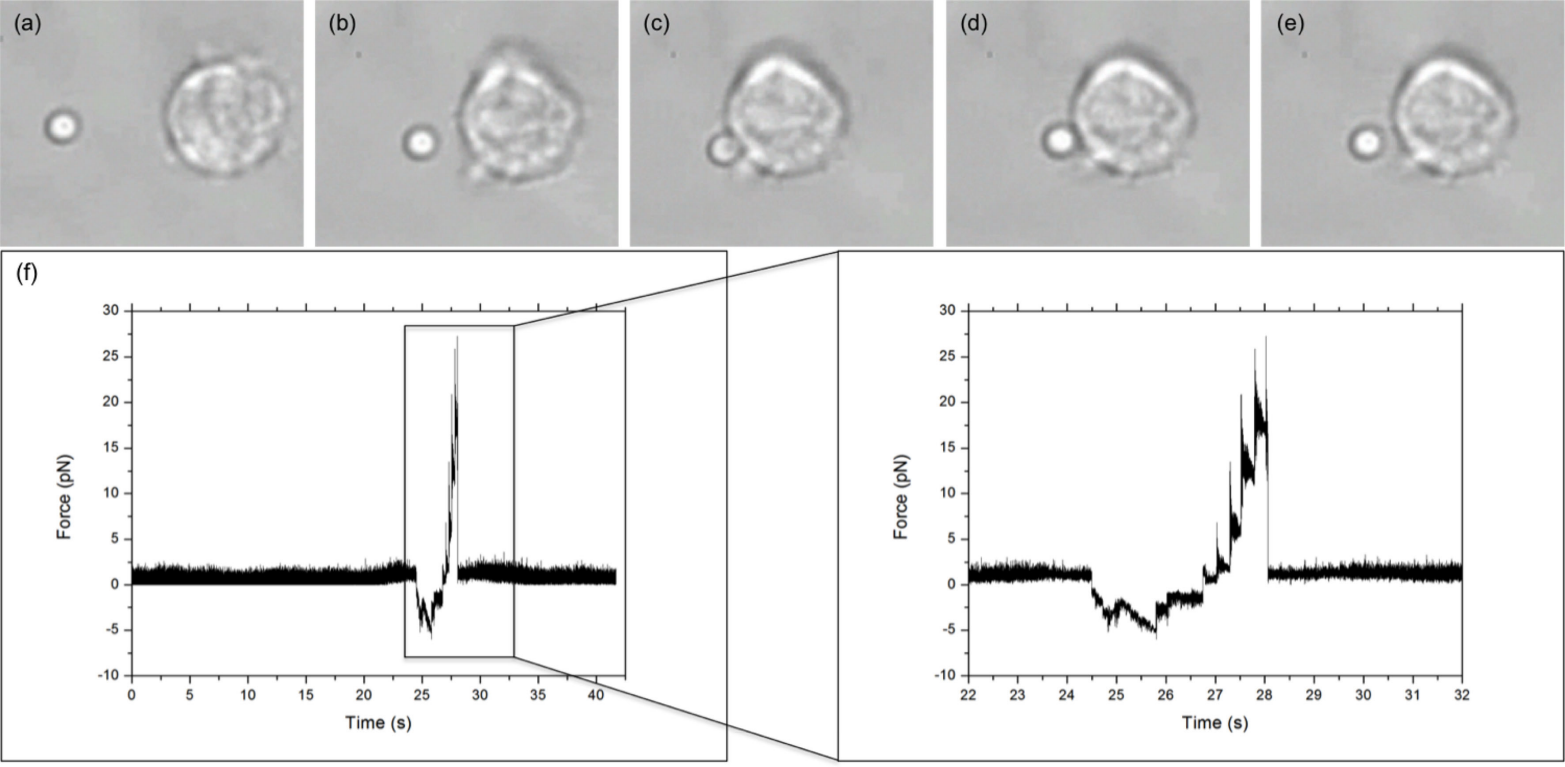
LFA-1–ICAM-1 adhesion measurement. **(A–E)** show the five steps of an adhesion measurement: **(A)** trap stiffness; **(B)** approach; **(C)** stick; **(D)** pull; and **(E)** bond break. Arrow donates direction of stage/cell movement. **(F)** Typical force versus time graph obtained for an integrin–ligand unbinding event. The large peak, enlarged in the inset, represents the maximum unbinding force required to disrupt an integrin–ligand bond. The jagged profile of the peak is due to the piezo stage moving in **“**steps.**”**.

The binding between cell and bead was measured using a five-step process, illustrated in **Figures 2A–E**, *via* LabVIEW. Before executing the program, a bead was trapped with ˜200 mW and positioned ˜10 *μ*m from a cell. Trap stiffness was determined by sampling the QPD at 100 kHz, in 4-s intervals for 20 s. Power spectra were calculated from the average signal of five intervals with the corner frequency, and hence trap stiffness, determined through a non-linear least square fitting of a Lorentzian function (14) (**Figure 1** inset). After determining trap stiffness, the cell and bead make contact, pushed together with ˜5 pN for 1 s, and then separated. While the data sampling rate from the QPD remained constant, the sample number was decreased to 8,000 samples per interval using the cell-bead approach, allowing for pseudo-real-time bead displacement monitoring. An integrin–ligand bond is broken when the bead jumps back into the centre of the trap, producing a similar QPD voltage trace to that of **Figure 2F**.

### 2.8 Single Bond Frequency

Three outcomes were possible when performing an adhesion measurement: the bead and cell did not stick; the bead and cell remained stuck together; or the bead would jump into the trap, breaking the bond with the cell. Successful adhesion events occurred with an average frequency of 25% for WT cells and 22% for KI cells. Due to Poisson distribution statistics (15, 16), this is indicative of a single LFA-1–ICAM bond probability for WT cells of 86.3%, while 12.4% will be double bonds and ˜1% will have 3 or more bonds (17). The slightly lower average binding frequency for KI cells gives bond probabilities of 88.7%, 10.5%, and ˜1% for, respectively, single, double, or three or more bonds.

## 3 Results and Discussion

**Figure 3:**
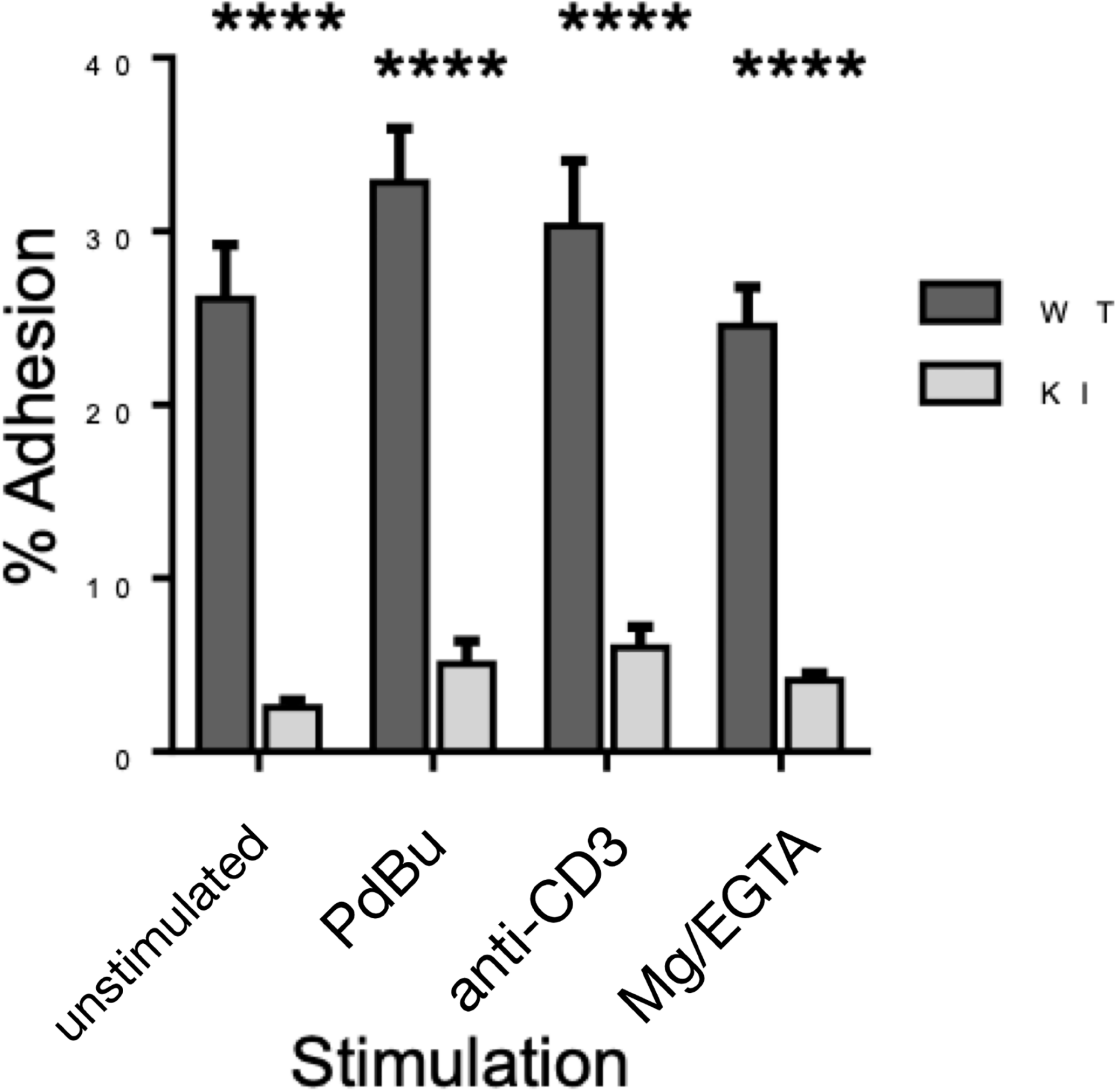
Adhesion of WT and KI cells to coated ICAM-1, as measured by static adhesion assay. Pooled data from 3 independent experiments, each with 2 technical replicates. ****p < 0.0001.

**Figure 4:**
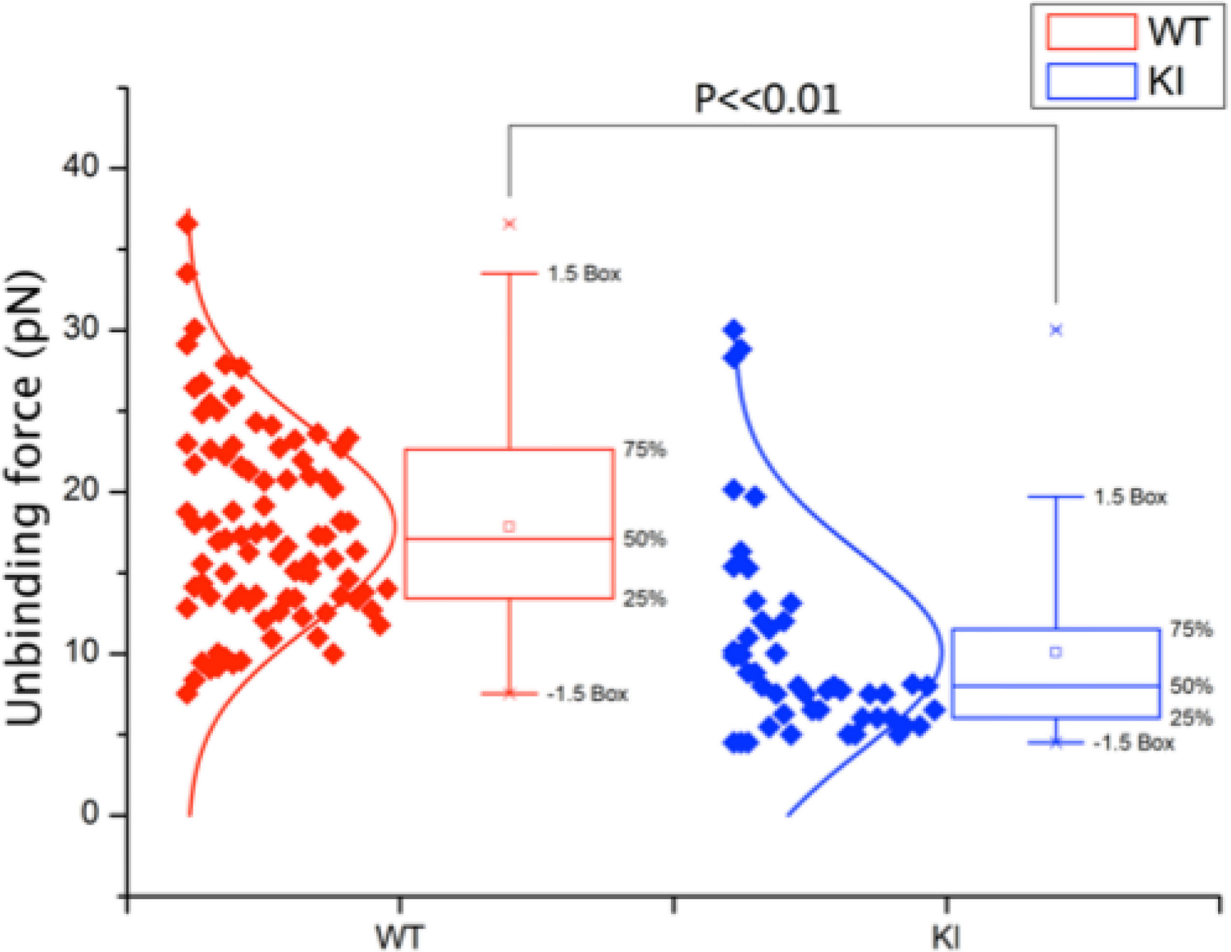
Unbinding force of integrin–ligand pairs for both WT and KI cells, as measured by an optically trapped 2.56-μm bead, coated with 2 μg/ml ICAM-1, in contact with the cell for 1 s. WT unbinding force was measured as 17.85 ± 0.63 pN (N = 91), with KI unbinding force significantly lower at 10.08 ± 0.88 pN (N = 49) (mean ± std. error, p << 0.01) at an average force loading rate on the cell of 30.21 ± 4.35 pN/s.

As we have previously shown (4), murine effector T cells display high spontaneous adhesion to coated ICAM-1 under static conditions, which could not be significantly upregulated with phorbol ester, anti-CD3, or Mg-treatment of the cells (**Figure 3**). Mutation of the kindlin-3 binding site in the integrin results in significantly reduced adhesion (**Figure 3**), as we have also previously shown with other immune cell types. However, how this mutation affects integrin–ligand bonds has remained unclear and has here been investigated utilizing optical trapping. The data presented in **Figure 4** show that LFA-1 on both WT and KI murine effector T cells can form spontaneous adhesive contacts to ICAM-1, its corresponding ligand, under force. An average integrin–ligand unbinding force of 17.85 ± 0.63 pN (mean ± std. error, N = 91) (most likely representing a single LFA-1-ICAM-1 bond) was measured for WT cells at an average force loading rate cell of 30.21 ± 4.35 pN/s. As expected, the unbinding force of the KI cells was significantly lower (p << 0.01, two tailed two-sample *t*-test), with an average unbinding force of 10.08 ± 0.88 pN (mean ± std. error, N = 49) at the same force loading rate. No bonds were measured as forming between uncoated silica beads.

The significant reduction of kindlin-3 binding to the integrin, achieved through the mutation of the TTT motif in the *β*2 integrin cytoplasmic domain, is mirrored in the reduction of rupture force of the integrin–ligand bonds when external force is applied. Integrins form so-called catch bonds, which are strengthened under force, until a limit is reached, breaking the bond. Under force, LFA-1 unbends from the closed conformation, allowing catch bond formation, as the applied force “pulls” the integrin open (18). The significantly lower rupture force of integrin–ligand bonds for the KI cells under force highlights the necessity of kindlin 3– *β*2 interaction for such catch bonds to form. Previous work by Morrison et al. showed that there was not a significant decrease in integrin expression of KI effector T cells (7); therefore, this is not a contributing factor for decreased integrin-mediated adhesion of these cells. However, Morrison et al. showed that, under shear flow (e.g., force), effector T cells with the TTT/AAA-mutated integrins were not able to form stable adhesion with ICAM-1 but were able to mediate cell rolling (7). Together with the results presented here, these results strongly indicate that the LFA-1–kindlin-3 interaction is necessary for LFA-1 catch bond formation.

**Figure 5:**
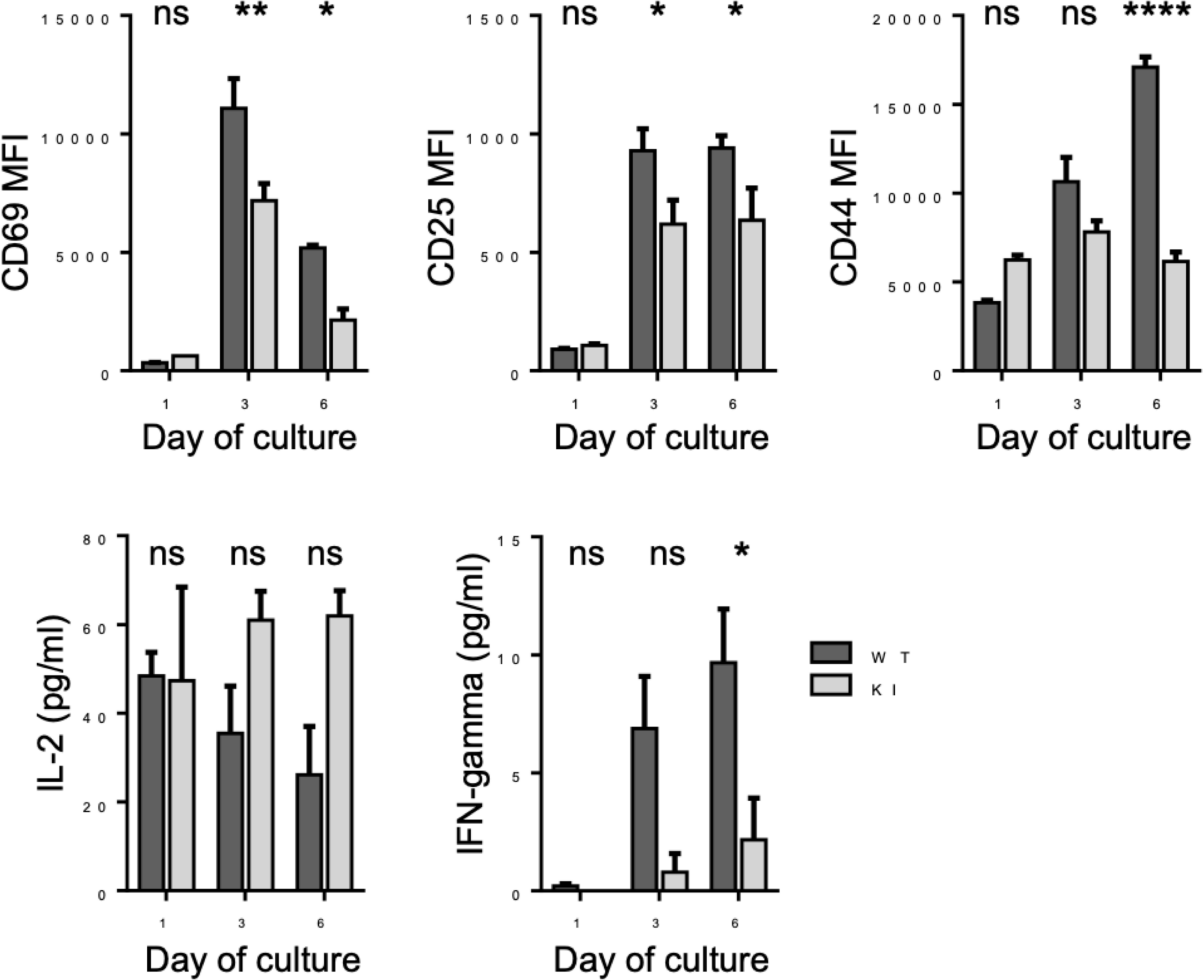
Activation of WT and KI CD8+ T cells following stimulation with anti-CD3 stimulatory antibodies for 1-6 days *in vitro*. Pooled data from 2 independent experiments, each with 2 biological replicates (donor mice). *p < 0.05; **p < 0.01; ****p < 0.0001; ns, non significant.

Integrin-mediated ligand binding is important not only for recruitment of T cells from the blood stream but also for other T cell functions. For example, optimal T cell activation is known to require LFA-1. To investigate whether the integrin/kindlin interaction, and therefore strong integrin–ligand bonds, is required also for CD8+ T activation, we performed T cell activation studies *in vitro*. Purified CD8+ T cells were activated *in vitro* utilizing soluble anti-CD3 antibodies, a mode of activation which requires functional integrins (7). During activation with soluble anti-CD3 antibodies, the T cells form cell–cell aggregates involving LFA-1–ICAM-1 bonds, which is necessary for optimal T cell activation. We therefore employed this T cell activation assay, to correlate LFA-1 bond strength with a biological outcome. Indeed, as shown in **Figure 5**, when the integrin/kindlin interaction is disrupted, T cell activation *in vitro* (e.g., expression of activation markers CD69, CD25, and CD44, and production of the cytokine IFNgamma, but not IL-2) is significantly reduced.

In conclusion, we have shown that kindlin 3– *β*2 integrin interaction affects integrin–ligand catch bonds of effector T cells and that firm adhesion of WT integrin–ligand pairs relates to an unbinding force of 17.85±0.63 pN at an average force loading rate on the cell of 30.21 ± 4.35 pN/s. By reducing the kindlin 3– *β*2 interaction, integrin–ligand unbinding force fell significantly, to 10.08±0.88 pN. This confirms that there is still an integrin–ligand interaction without kindlin-3 binding but that kindlin-3 indeed is required for optimal integrin–ligand catch bond formation.

How might kindlin-3 affect LFA-1 catch bond formation? Integrin activation can be achieved by extracellular forces but, in addition to this, also by cytoskeletal forces (pulling on the integrin from the inside of the cell) (19). LFA-1 on the surface of effector T cells is not in an active conformation (20), but effector T cell adhesion to ICAM-1 under shear flow, e.g., catch bond formation, requires an intact actin cytoskeleton (4). Interestingly, the TTT/AAA mutation in the integrin tail disrupts the interaction of the integrin with the actin cytoskeleton (21). We therefore postulate that kindlin-3 affects LFA-1 catch bonds by indirectly mediating its connections with the actin cytoskeleton within the cell. These interactions would allow anchoring LFA-1 within the cell, and catch bond formation in the presence of ligand.

We note that there is still significant scope to expand the observed results in terms of exploring behaviour as a function of loading rate and also experiments with changes in ligand concentration to clarify the nature of the single and multiple bond response.

This study brings new information about the role of kindlin-3 in regulating LFA-1-mediated T cell adhesion events necessary for proper immune system function, through allowing for LFA-1 catch bond formation. In the future, it will be interesting to expand these studies to examine the exact mechanisms involved in kindlin-3-regulated LFA-1 binding to ICAM-1 in T cells, as well as in other cell types.

## Data Availability Statement

The original contributions presented in the study are included in the article/supplementary material. Further inquiries can be directed to the corresponding author.

## Ethics Statement

The animal study was reviewed and approved by the University of Dundee ethics committee.

## Author Contributions

SF and DM designed the study, CM and VM performed experiments and analysed data, CM, VM, DM, and SF wrote the manuscript. All authors contributed to the article and approved the submitted version.

## Funding

This study was funded by the Engineering and Physical Sciences Research Council (EPSRC) EP/J500392/1; Academy of Finland (to SF); and Ella and Georg Ehrnrooth Foundation (to VM).

## Conflict of Interest

The authors declare that the research was conducted in the absence of any commercial or financial relationships that could be construed as a potential conflict of interest.

## Publisher’s Note

All claims expressed in this article are solely those of the authors and do not necessarily represent those of their affiliated organizations, or those of the publisher, the editors and the reviewers. Any product that may be evaluated in this article, or claim that may be made by its manufacturer, is not guaranteed or endorsed by the publisher.
